# Deep learning-driven proteomics analysis for gene annotation in the renin-angiotensin system

**DOI:** 10.1016/j.ejphar.2025.178119

**Published:** 2025-09-02

**Authors:** Mortaza Eivazi, Kamran Hosseini, Shahin Alipanahi, Huijing Xia, Luke Restivo, Ayushi Patel, Mahdieh Gozali, Tahereh Ebrahimi, Amy Scarborough, Vahideh Tarhriz, Eric Lazartigues

**Affiliations:** a Department of Computer Science, Faculty of Mathematics, Statistics, and Computer Science, University of Tabriz, Tabriz, Iran; b Shiraz Neuroscience Research Center (SNRC), Shiraz University of Medical Sciences, Shiraz, Iran; c Cardiovascular Center of Excellence, Louisiana State University Health Sciences Center, New Orleans, LA, 70112, USA; d Department of Pharmacology & Experimental Therapeutics, New Orleans, LA, 70112, USA; e Molecular Medicine Research Center, Tabriz University of Medical Sciences, Tabriz, Iran; f Southeast Louisiana Veterans Health Care System, New Orleans, LA, 70119, USA

**Keywords:** Renin-angiotensin system, Multi-layer perceptron, Hypertension, Machine learning

## Abstract

The renin-angiotensin system (RAS) is central to cardiovascular diseases such as hypertension and cardiomyopathy, yet the functions of many RAS genes remain unclear. This study developed a multi-label deep learning model to systematically annotate RAS gene functions and elucidate their roles in biological pathways. A total of 39,463 RAS-related publications from PubMed and PMC were processed into text format. Feature matrices were generated using TF-IDF and token processing, followed by dimensionality reduction via Principal Component Analysis (PCA). A Multi-Layer Perceptron (MLP) was applied for multi-label classification, with performance evaluated using Precision, F1-Score, Ranking Loss, and ROC-AUC metrics. The model outperformed traditional methods (SVM, Random Forest), achieving a Precision of 0.7474 and ROC-AUC of 0.8697. Grouping into three major biological branches improved interpretability and performance (Precision: 0.8312; ROC-AUC: 0.9182). *In silico* predictions were validated using extracellular vesicle (EV) proteomics and capillary Western assays in DOCA-salt hypertensive mice. Key genes-*AGTR2, IRAP (LNPEP), Ywhas (SFN), EDNRA*, and *ESR2*—were identified as critical RAS components. Notably, IRAP was markedly upregulated in hypertension and showed regulatory interactions with 14-3-3 proteins, modulating Nedd4-2, ACE2, and AGTR1 signaling. To our knowledge, this is the first integration of multi-label AI modeling with EV proteomics for RAS pathway annotation. This framework captures complex gene-pathway relationships, advancing systems-level understanding of RAS biology and revealing a novel IRAP/Ywha(s)/Nedd4-2–ACE2 interaction axis as a potential therapeutic target.

## 1. Introduction

Despite the accumulation of a significant body of knowledge, the field of cardiovascular research requires new targets, as evidenced by the approval of 55 novel drugs in 2023, emphasizing ongoing efforts to address cardiovascular health (Mullard, 2024). The renin-angiotensin system (RAS) is critical for regulating blood pressure and fluid balance, with dysregulation linked to cardiovascular diseases such as hypertension, diabetic cardiomyopathy hypertrophic cardiomyopathy, as well as COVID-19 (Batista et al., 2023; Bogdarina et al., 2007; Rysz et al., 2021). Angiotensin-II (Ang-II) is a key peptide hormone in the RAS and exerts its effects through the Ang-II type 1 receptor (AGTR1/AT_1_R), leading to vasoconstriction, sodium reabsorption, and increased blood pressure (Mendoza and Lazartigues, 2015). While significant research has elucidated key components of the RAS, many gene functions within this system remain incompletely understood. Recent research has uncovered additional RAS axes, such as the ACE2/Ang-(1–7)/Mas and the Ang-IV/IRAP (insulin-regulated aminopeptidase), which play crucial roles in physiological and pathological processes and expand the functional diversity of the system (Härdtner et al., 2013; Xu et al., 2011). Ang-IV/IRAP represents a functional signaling mechanism involving Ang-IV and Leucyl/cystinyl aminopeptidase (LNPEP), also known as IRAP, which influences various physiological processes, particularly in the brain and cardiovascular system (Balakumar et al., 2022). Given the complexity of the RAS and the related pathways involved in hypertension and cardiovascular diseases (CVDs) and the exponential growth of biomedical literature, manual annotation of gene functions has become increasingly challenging. (Hirschman et al., 2012; Ji et al., 2017; LeCun et al., 2015). Advancements in artificial intelligence, particularly deep learning, have transformed biomedical research by enabling automated extraction of meaningful patterns from large datasets (Lau et al., 2023). Deep learning algorithms offer a solution to enhance the accuracy and efficiency of gene function annotation. These algorithms are efficient in analyzing high-dimensional data and identifying complex patterns, making them suitable for bioinformatics applications (Hirschman et al., 2012; Kanehisa et al., 2016). However, existing gene annotation approaches often rely on single-label classification, limiting their ability to capture the multi-functional roles of genes within complex biological systems such as the RAS (Liu et al., 2022). This study aims to develop a multi-label gene function annotation model for the RAS using deep learning techniques. By analyzing full-text biomedical publications, the model facilitates a comprehensive understanding of gene functions and their roles within the RAS. Multi-label learning is especially suitable for this task, as genes often participate in multiple biological pathways, each associated with different functions. The proposed model captures these complex relationships, providing more accurate and detailed annotations than traditional methods.

## 2. Materials and methods

### 2.1. Data Collection and feature matrix construction

Genes within the RAS and their interactions were annotated in the pathway plots and the orthologous gene annotations were revised by the UCSC Genome Browser (Karolchik et al.), (https://genome.ucsc.edu). The completed revision was performed in the network of protein-protein interactions for a better understanding of the coherence between various molecules that lead to the biological activities of the RAS and its related pathways. The protein-protein interactions and orthologue associations for involved molecules, particularly ACE2 in the RAS, were anticipated using the Search Tool for Retrieval of Interacting Genes and Proteins, codenamed STRING (Szklarczyk et al., 2017) (https://stringdb.org/). For understanding high-level functions and utilities of the RAS, in the cell and organism using molecular-level information, in particular large-scale molecular datasets generated by the mentioned tools, we used KEGG, GenomeNet (Kanehisa et al., 2002) (https://www.genome.jp/kegg/genes.html). All information on genes in the mentioned pathways, their functions, and all probable interactions were extracted using Gene IDs as KGML files (Guan et al., 2018). Then, all commonly used names for every gene in the mentioned pathways were collected. Afterward, all publications about the RAS, and the related pathways (nine pathways) that are available in the NCBI database were gathered using the Entrez e-utility tool and their PubMed IDs (https://pubmed.ncbi.nlm.nih.gov/), covering the gene functions, were fetched. We conducted our systematic research in accordance with the PRISMA guidelines (Moher et al., 2009). A literature search was conducted to in the electronic databases of PubMed by screening “Free full text”. To ensure coverage of all relevant aspects of the RAS, we followed PRISMA guidelines and conducted our search using standardized terms in PubMed and PMC. Inclusion Criteria including **Study Focus:** Renin secretion/Aldosterone synthesis and secretion/Aldosterone-regulated sodium reabsorption/Vasopressin-regulated water reabsorption/Estrogen signaling/Diabetic and hypertrophic cardiomyopathy; **Search Terms:** “renin and/or (−) angiotensin system”, “Renin secretion”, “Aldosterone synthesis and/or secretion”, “Diabetic cardiomyopathy renin and/or (−) angiotensin system”, “Hypertrophic cardiomyopathy renin and/or (−) angiotensin system”, “Aldosterone and/or () regulated sodium reabsorption renin and/or (−) angiotensin system”, “Vasopressin-Regulated Water Reabsorption signaling pathway renin and/or (−) angiotensin system”, “Dilated cardiomyopathy renin and/or (−) angiotensin system”; Availability: **Format:** Publications with available PubMed ID and PMC ID, enabling reliable downloading and processing, **Language:** Articles published in English; C**ontent Type:** Peer-reviewed original research articles, reviews, and data-focused reports relevant to gene function within RAS pathways. Both the PubMed ID and PMC ID of obtained publications were downloaded via PMC FTP Service. Before feeding the data into the model, we checked for outliers, missing values, duplicates, and verified the data type of each variable to ensure basic data integrity and type safety. Additionally, we addressed class imbalance by applying weighted classes to prevent bias toward dominant conditions; and performed feature selection using the Principal Component Analysis (PCA) method (Tonin et al., 2024) to eliminate irrelevant features and reduce dimensionality. Text cleaning and normalization converted abbreviations, typos, and non-standard language into standard terms. Briefly, we excluded articles that lacked a focus on the RAS or its associated pathways, those without gene- or pathway-specific information, non-English publications, paywalled articles without accessible content, entries missing essential metadata such as PubMed ID or PMC ID, and low-quality or incomplete texts. These preprocessing steps were essential for extracting accurate and unbiased data from PubMed to ensure reliability of the multi-label classification task.

### 2.2. Deep learning model implementation and model evaluation

After downloading 39,463 PDF publication files, they were converted into text files to be mined appropriately. For feature extraction, initially, we used pre-preprocess methods on the downloaded data to enhance data quality and eliminate noise and inconsistencies, thus optimizing the model’s performance. At the first step, we tokenized the texts, which is a method to break down a text into individual tokens alongside removing any extra components such as punctuations and stop words to better process our articles. For the final step of the feature extraction, we used TF-IDF, which is a statistical method used in natural language processing and information retrieval to measure the importance of a word within a document relative to a collection of documents for a specific gene name.

- $\text{TF}=TF(t,d)=\frac{f_{t,d}}{\sum_{t^{\prime }\in d}f_{t^{\prime },d}}$
  - $f_{t,d}$: Number of times term t appears in document d.
  - Denominator: Total number of terms in document dd.
- $\text{IDF}=IDF(t,D)=\text{log}\;\left(\frac{N}{n_{t}}\right)$
  - N: Total number of documents in the corpus.
  - $n_{t}$: Number of documents containing the term tt.
  - **Logarithm** (base ee, 2, or 10) scales the value to reduce skew from large corpora.
-
TF-IDF(t,d,D)=TF(t,d)*IDF(t,D)

(i) TF calculates the term frequency of a word in an article. (ii) The IDF was calculated by taking the logarithm of the total number of documents divided by the number of documents containing the term. (iii) We calculated the product of TF and IDF to retrieve the final measurement. While there are multiple gene names for a particular gene, after calculating the TF-IDF score for all the names, we returned the value for the corresponding gene ID alongside the previous annotation for the gene as labeled giving a 1026 × 39,463 feature matrix.

Several learning algorithms, such as SVM using the One-vs-Rest classifier, Random Forest, XGBoost, and Back-Propagation Multi-Label Learning, were trained to predict the gene multi-function based on the collected data matrix. The MLP with the PCA model was selected as the deep learning algorithm. The feature matrix consisting of 1026 gene names and 39,463 gene-related full-text publications were fetched from PMC FTP services. To optimize the model’s performance, dimensionality reduction techniques were applied to decrease the complexity of the dataset, effectively reducing the training time while preserving essential features of the data for accurate learning. To annotate gene functions, we implemented a multi-label deep learning model comprising a Principal Component Analysis and an MLL (multi-label learning) framework.

### 2.3. Dimensionality reduction: Principal Component Analysis (PCA)

Overfitting happens when a model learns unnecessary patterns from excessive features, reducing its ability to generalize. To mitigate this, PCA was applied to transform correlated variables into fewer uncorrelated components while preserving critical dataset characteristics. This reduces redundancy, improves efficiency, and ensures better model performance on new data. After the standardization of the data, the steps to implement the PCA were as follows:

1. *Covariance Matrix:* The Covariance Matrix aims to find the relationship between distinct features by calculating the equation below:
$$\Sigma =\frac{1}{n-1}Z^{T}Z$$
  Where the Σ is the covariance matrix, n is our sample, and $Z^{T}$ is the transpose of the standardized Feature matrix Z.
2. *Computation of Eigenvectors and Eigenvalues:* Find the eigenvectors and eigenvalues leading in order to calculate the directions and their importance.
Σv=λv
  Where v is an eigenvector and λ is the corresponding eigenvalue.
  Furthermore, we sorted the eigenvalues in descending order and arranged the eigenvectors to create the principal components.
3. *Select the principal components and transform the data:* Choose the top k eigenvectors corresponding to the k largest eigenvalues value the k-dimensional feature subspace. After this, we were able to project the initial standardized dataset into k-dimensional subsets through the equation below:
$$T=ZV_{k}$$
  Where the T is transformed data and $V_{k}$ is the matrix containing the selected eigenvectors.

#### 2.3.1. Model performance optimization through pathway category reduction

To achieve a higher F1-score, the ten pathways were further categorized into three general branches: hypertension-related pathways, cardiac disease-related pathways, and diabetes-related pathways. Hypertension-related pathways are directly or indirectly involved in blood pressure regulation and salt/water balance. Cardiac disease-related pathways are associated with structural and functional abnormalities of the heart. Diabetes-related pathways contribute to signaling mechanisms and complications associated with diabetes. It is worth noting that diabetic cardiomyopathy may also overlap with the cardiac disease-related group due to its impact on cardiac structure and function.

### 2.4. Hypertension mouse model

Regarding the RAS and its intricate interplay, we aimed to validate our *in-silico* findings through an experimental model, the DOCA-Salt hypertensive mouse (Basting and Lazartigues, 2017; Hilzendeger et al., 2013). Experiments were conducted in adult C57BL6/J mice (11–16 weeks old, 20–30 g; Jackson Laboratory, Bar Harbor, ME) from both sexes. Mice were housed in a temperature (~25 °C) and humidity-controlled facility under a reversed 12-h dark/light cycle, fed standard mouse chow (Envigo, iOS Teklab Extruded Rodent Diet, 2019S, Huntingdon, UK) and water ad libitum. All procedures conformed to the National Institutes of Health Guide for the Care and Use of Laboratory Animals and were approved by the Louisiana State University Health Sciences Center (#3873), and the Southeast Veterans Healthcare System (#620) Institutional Animal Care and Use Committees in accordance with the ‘Principles of Laboratory Animal Care by the National Society for Medical research and the Guide for the Care and Use of Laboratory Animals’ (National Institutes of Health Publication No. 86–23, revised 1996). The animal study protocol was approved by the Institutional Animal Care and Use Committee (IACUC) of University of LSU Health Sciences Center (Protocol #: 7119). Briefly, mice (n = 20–25 per groups) were anesthetized with isoflurane (2–3 %) in an oxygen flow (1 L/min) and placed on a heating pad to maintain body temperature around 37.5 °C. Buprenorphine-SR (1 mg/kg, sc) was injected before any surgery for pain relief. Male and female mice were subjected to uni-nephrectomy during which an incision was made in the skin in the retroperitoneal region to remove the right kidney. After 1 week of recovery, mice were implanted subcutaneously with a DOCA pellet (50 mg, 21-day release, Innovative Research of America, Cat #: M-121) or underwent sham surgery. Drinking water from DOCA-implanted mice was replaced with 1 % NaCl solution. Prior to DOCA implantation, blood (100–200μL) was collected from the facial vein under light isoflurane anesthesia, followed by centrifugation to separate plasma. Three weeks after DOCA-salt treatment, mice were sacrificed by decapitation, and plasma samples were collected. Samples from each group were pooled for extracellular vesicles proteomics analysis.

#### 2.4.1. Extracellular Vesicles (EVs) protein extraction and LC-MS analysis

The extraction and quantification of the EVs’ proteins was carried out by Creative Biolab Inc. (Project ID: CBLK033123–1c-E). Briefly, plasma samples were thawed, pooled, before EV extraction. EV’s proteins were extracted using magnetic beads, followed by lysis with 7M urea and 2 % SDS. After centrifugation, the supernatant was collected, and protein concentration was determined using the BCA assay. For digestion, 30 μg of protein was reduced with DTT (10 mM), alkylated with IAA (DTT:IAA = 1:5), washed with acetone, and digested overnight with trypsin. Peptides were desalted using a Monospin column, eluted with 50 % acetonitrile, dried, and reconstituted in 0.1 % formic acid. Samples (1–2 μg) were analyzed on an EASY-nLC 1200 coupled to an Orbitrap Exploris 480+FAIMS Pro mass spectrometer (Thermo Scientific). Data were acquired in Data-Dependent Acquisition (DDA) mode (m/z 350–1600) with 60,000 resolution and HCD fragmentation at 30 % collision energy.

### 2.5. Capillary western analysis

Total protein lysates were extracted from plasma samples collected from male (MD) and female (FD) DOCA-Salt hypertensive mice and their respective controls (n = 5–7, aged = 3–6 months). The following antibodies were used for immunodetection: rabbit polyclonal anti-CD-249 antibody (ThermoFisher, Cat# PA5–88487), rabbit polyclonal anti-CD-13 antibody (ThermoFisher, Cat # MA5–32311), rabbit polyclonal anti-LNPEP antibody (ThermoFisher, Cat# PA5–23777), Ywhah ThermoFisher, Cat# 15222–1-AP), Ywhaq (ThermoFisher, Cat# PA5–17426), and goat anti-rabbit secondary antibody. Protein concentrations were determined using a BCA assay (Cortés-Ríos et al., 2020). The protein samples were denatured by heating at 95 °C for 5 min, and 3μL of each sample was loaded into individual wells of the capillary cartridge. The cartridge was preloaded with separation matrix, stacking matrix, and detection reagents according to the manufacturer’s protocol. Protein concentrations were determined using a BCA assay (source), and samples were prepared by mixing with 0.1X sample buffer. The samples were denatured by heating at 95 °C for 5 min, and 3μL of each protein sample was loaded into the respective wells. The capillary cartridge was loaded with separation matrix, stacking matrix, primary and secondary antibodies, protein samples, and detection reagents, following the ProteinSimple system’s manual (https://www.bio-techne.com/brands/proteinsimple). Signal intensities were quantified based on the total proteins, and the aminopeptidases expression levels across different samples were compared using the system’s analysis software.

### 2.6. Statistical analysis

R language and RStudio (https://rstudio.com/) were utilized as an integrated development environment (IDE), and Python and Visual Studio Code (https://code.visualstudio.com) were used as additional IDEs for RNAseq data analysis. Data is presented as the mean ± standard error of the mean (SEM). Statistical differences between groups were determined using a t-test, one-way ANOVA, and post-hoc Tukey with a significance threshold set at p ≤ 0.05. All analyses were conducted using GraphPad Prism 8 (GraphPad Software, San Diego, CA, USA).

## 3. Results

### 3.1. Model performance for gene function prediction

To annotate gene functions within the RAS using a multi-label deep learning approach, we collected relevant and freely available biomedical publications from the PubMed and PMC databases. Specific search strings related to the RAS and its associated pathways were employed to filter the articles, ensuring that they included both PubMed IDs and PMC IDs for full-text access. This resulted in the acquisition of approximately 39,463 publications, which were subsequently converted into text files for analysis. To validate the generalization of our models, two methods were conducted to assess the model’s capabilities. Since there is a limited number of genes in our pathway, we tried a 10 k-fold cross-validation to assess the model’s prediction ability. Secondly, we split our data into an 80 % train set and a 20 % test set.

We compared the performances of MLP, Support Vector Machine (SVM) with the One-vs-Rest method, Random Forest, and XGBoost algorithms altogether. SVM aims to determine a hyperplane in a high-dimensional space to classify the records while simultaneously trying to maximize the margin between them. For multi-class problems, the One-vs-Rest (OvR) strategy trains one binary SVM classifier per class, each separating that class from all others. The Random Forest Algorithm is a classification model that utilizes a cluster of decision trees, each trained on random subsets of the data and features to ascertain one of the multiple categories through majority voting. Similarly, XGBoost uses an ensemble of decision trees, which is a decision support tool that employs recursive partitioning to create a tree-like model, illustrating decisions and their potential outcomes. Additionally, XGBoost corrects the error of the previous decision trees in a sequence through gradient boosting and regularization methods, making it a powerful tool for both regression and, in this case, a multi-label task. However, the MLP uses a loss function to calculate the errors of the model before trying to optimize it and minimize the loss function’s value through back propagation (Tables 1 and 2). The loss function measures the difference between a model’s predictions and the actual outcomes, calculating the model’s overall error.

We have adopted four metrics for the conducted models which are ranking loss and Macro average forms of Precision, F-1 Score, and ROC-AUS. Macro-averaging treats each class equally, regardless of how many samples it has.

Suppose we have K = 11 classes representing the genes. For each class i∈{1,…,K}, we define a one-vs-rest binary classification problem (class I vs. all other classes) and compute the standard binary metrics.

Precision evaluates the number of correct true positive conjectures divided by the sum of true positives and false positives.

$$P_{i}=\frac{TP_{i}}{TP_{i}+FP_{i}}\left(if\right.\;TP_{i}+FP_{i}>0,\text{else}\;\text{define}\;\left.P_{i}=0\right),{\text{Perecision}}_{\text{macro}}=\frac{1}{K}\sum_{i=1}^{K}P_{i}$$

Where TP stands for True Positive, which are the samples that we have correctly classified, and FP stands for False Positive, which are the samples that we have mistakenly labeled positive.

Recall (also known as true positive rate) measures how well a classifier identifies all of the positive instances.

$$R_{i}=\frac{TP_{i}}{TP_{i}+FN_{i}}\left(if\right.\;TP_{i}+FN_{i}>0,\text{else}\;\text{define}\;\left.R_{i}=0\right),{\text{Recall}}_{\text{macro}}=\frac{1}{K}\sum_{i=1}^{K}R_{i}$$

The next metric is the F-1 score, which calculates the mean harmonic of Precision and Recall, ensuring that the model does not favor one over another making F-1 Score a robust metric when False positives and False Negatives must be minimized.

$$F_{1,i}=2\frac{P_{i}R_{i}}{P_{i}+R_{i}}\left(if\right.\;P_{i}+R_{i}>0,\text{else}\;\left.F_{1,i}=0\right),F_{1,\text{macro}}=\frac{1}{K}\sum_{i=1}^{K}F_{1,i}$$

Ranking loss measures the penalty for disordering pairs of items; more specifically, it compares the predicted scores from the model with true relevance labels.

$$\text{Ranking}\;\text{Loss}=\frac{1}{\left|P\right|}\underset{(i,j)\in P}{\sum }\text{ln}\left(1+e^{-\left(s_{i}-s_{j}\right)}\right)$$

Finally, ROC AUC measures the performance of a binary classifier across all classification thresholds, evaluating how well the model distinguishes between two positive and negative classes. More specifically, ROC plots the True Positive Rate against the False Positive Rate at varying thresholds coupled with AUC, which is the area under the ROC curve. ROC AUC consists of several steps:

1. True Positive Rate/Recall:
$$TPR=\frac{TP}{TP+FN}$$
2. False Positive Rate:
$$FPR=\frac{FP}{FP+TN}$$
3. $\text{AU}{\text{C}}_{\text{i}}$
$$AUC_{i}=\int_{0}^{1}TPR_{i}(t)d\left(FPR_{i}(t)\right)(\text{area under the}\;ROC\;\text{curve}),AUC_{\text{macro}}$$
$$=\frac{1}{K}\sum_{i=1}^{K}AUC_{i}$$
  Where P stands for the number of positive samples and N stands for the number of negative samples. (1026 distinct genes are in ten pathways, meaning that we have a small amount of data).

In such circumstances, it becomes challenging to train the model precisely without overfitting. Nonetheless, we tested the generalization of the models in two ways. First, we split data into a 90 percent train set and a 10 percent test set. We then implemented a 10-fold cross-validation on 90 percent of the data. During this experiment, the 90 percent is divided into 10 separate subsets with one subset serving as a validation in each assessment. Afterward, we tried to test the model performances on the remaining test set. The second column (precision metric) of Tables 1 and 2 highlights that Random Forest outperforms other approaches with 0.79 precision on the cross-validation section, while the MLP achieves the second-best result with 0.7474 Precision. However, for the test results, XGBoost has the highest Precision of 0.9524, while the MLP scores the least Precision with a score of 0.6829. The third column (F-1 Score metric) of Tables 1 and 2 show the highest result of cross-validation, being 0.5846, output by MLP. Sequentially, SVM gets 0.3041 as the second-best performer in cross-validation. However, the MLP achieves the highest score of 0.6120 in the Test Results, while SVM achieves the second-best F1-Score of 0.5033. The fourth column (Ranking Loss metric) of Tables 1 and 2 indicate that MLP is the model with the best result among cross-validation results with a Loss of 0.1096. Next, the SVM model achieves the second-best result in the cross-validation section with a Loss of 0.1810. Nonetheless, Random Forest performs the best with a Loss of 0.1613 in the Test sets. Secondly, SVM comes next with a Loss of 0.1703. The fifth column (ROC-AUC metric) of Tables 1 and 2 indicate MLP to be the model with the most ROC-AUC with 0.8697 in the cross-validation sets. Additionally, SVM results in ROC-AUC of 0.8102 in second place in the cross-validation sets. Accordingly, in alignment with the cross-validation results, the test set also achieves the highest ROC-AUC, of 0.8528 on MLP and ROC-AUC of 0.8435 on SVM.

**Fig. 1.**
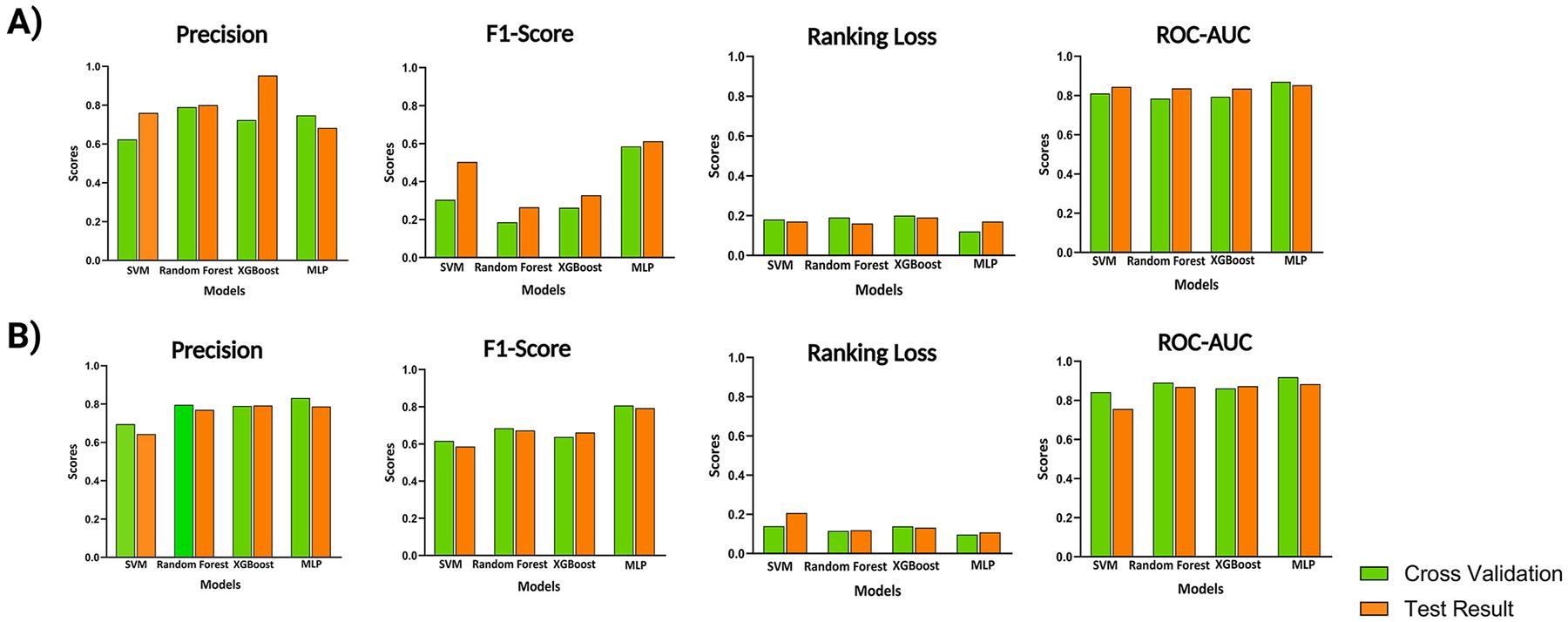
Comparison of machine learning model performance across evaluation metrics. Panels A and B present the results of four models-Support Vector Machine (SVM), Random Forest, XGBoost, and Multi-Layer Perceptron (MLP)-evaluated using Precision, F1-Score, Ranking Loss, and ROC-AUC metrics. For each model, results from cross-validation (green bars) and independent test sets (orange bars) are shown. (**A**) Performance on the dataset containing 10 pathways. (**B**) Performance on the dataset after categorizing the 10 pathways into three branches.

According to Fig. 1A, models other than MLP produce better Precision, particularly on Test Sets such as XGBoost with a score of 0.9524. They lack the desired F-1 Score, signaling that lower Recall values are present in both the cross-validation and Test Assessments. Therefore, many False Negative results are produced by those models. Whereas the MLP model performs well on both sets coupled with adequate Precision scores on Test Results. While other models may conclude a better Precision on the Test sets, they are less appealing on F-1 Metrics within both sets. As a result, the MLP provides better overall precision and F-1 Score compared to other models. MLP does a great job at keeping the Ranking Loss as low as 0.1096 in the cross-validation sets, which is considerable compared to other models. MLP also shows promising results, just behind Random Forest and SVM in the test set, with a Ranking Loss of 0.1765. According to Fig. 1A, all the models perform relatively well on ROC-AUC metrics. However, the MLP outperforms every single one in both cross-validations, with a value of 0.8528 and Test sets with a value of 0.8435. Furthermore, by categorizing the ten pathways into three branches, this restructuring led to an improvement in the F1-score from 0.6120 to 0. 8058 (Tables 3 and 4, and Fig. 1B).

### 3.2. Pathway-level gene predictions

**Fig. 2.**
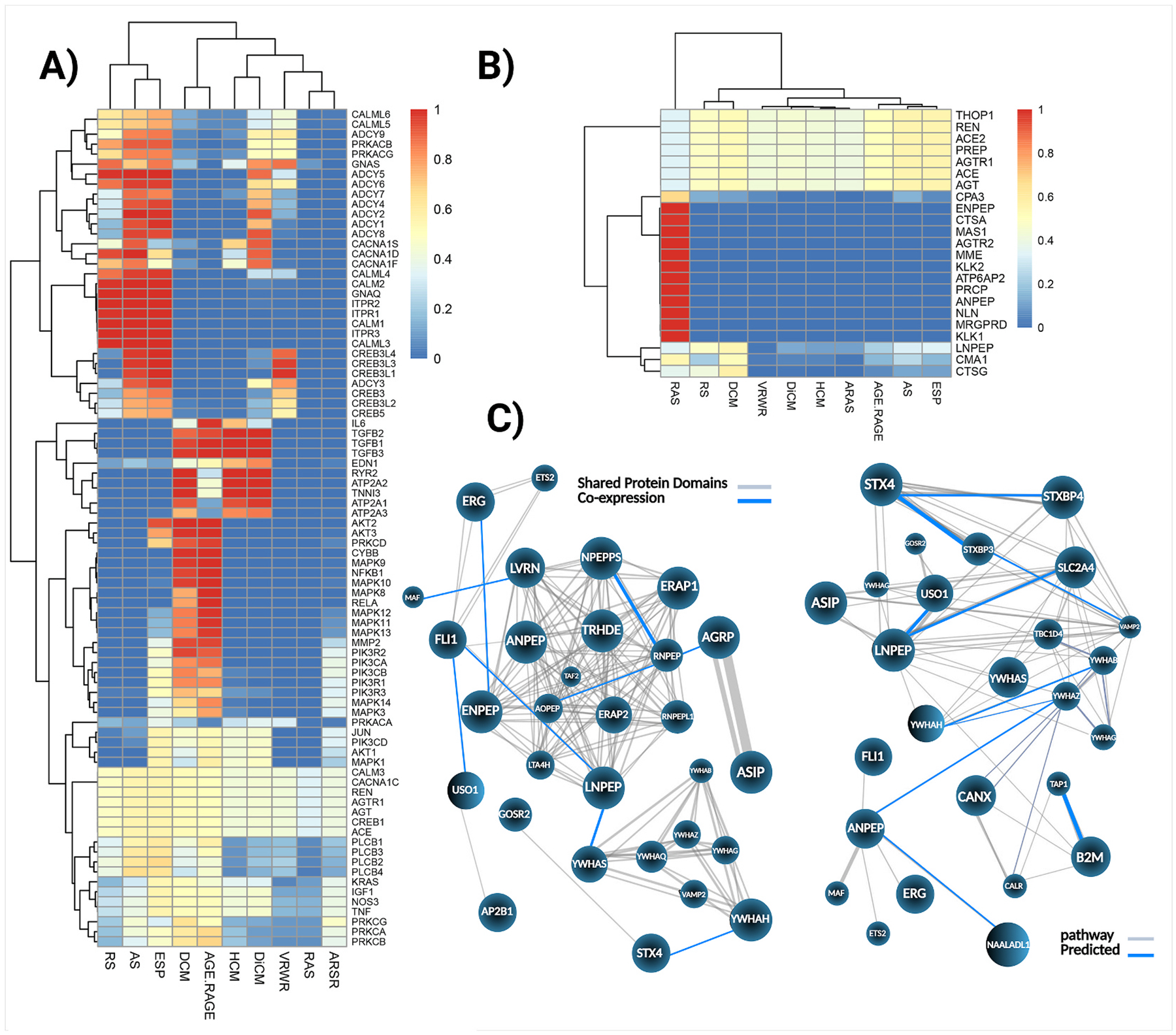
Multi-level analysis of hypertension-related genes and pathway networks. (**A**) pHeatmap showing hierarchical clustering of genes involved in blood pressure regulation, angiotensin signaling, and associated pathways across key KEGG terms, including RAS and various related cardiomyopathy and hormone signaling pathways. Gene expression levels are normalized and color-coded from low (blue) to high (red). (**B**) pHeatmap representing enriched pathway-level scores for RAS-related genes across KEGG pathways. Each column denotes a pathway, and each row indicates a RAS gene; the intensity of red reflects higher enrichment scores x = 1. (**C**) cNETplot illustrates functional interaction networks between LNPEP/IRAP and other genes. Edges indicate predicted associations based on shared protein domains (gray lines) and co-expression (blue lines).

To shorten the training time of our learning model, the data dimension of the problem was decreased as much as possible. The result is a novel annotation model using a deep learning method for gene multi-function discovery of the RAS and its relevance to specific pathways. This machine enhances the accuracy and speed of biological result analysis. According to the dataset description, all collected articles highlight specific gene functions, providing evidence for determining whether a gene is involved in a particular pathway. The most important gene was detected based on the Max Predicted Probability (Table 5). Our model identified the endothelin receptor type A (*EDNRA*) gene as a key gene associated with the RAS, playing a central role in blood pressure regulation, while estrogen receptor beta (*ESR2*) was highlighted in the estrogen signaling pathway for its specific involvement in hypertension. Additionally, *Ywhas (SFN/14-3-3 sigma/Stratifin*) emerged as the most significant molecule associated with the Aldosterone-Regulated Sodium Reabsorption pathway. Our findings indicate that AGTR2 may play a key role similar to AGTR1 in the RAS pathway, underscoring its significance as a key regulator in hypertension (Table 5). Subsequently, we selected genes with a score of x ≥ 0.9 that were shared among three or more pathways. The analysis revealed that *AGT*, *ACE*, *AGTR1, REN*, *CREB1*, *CALM3*, and *CACNA1C* were consistently present across all examined pathways (Fig. 2A). Additionally, we identified genes that were present in the RAS based on the KEEG pathway database (map04614) and compared their presence across other pathways. Seven of these genes appeared to function in multiple pathways, while twelve were found to be specific to the RAS pathway alone. Interestingly, *THOP1* has been identified as a gene potentially involved in pathways related to blood pressure regulation. This zinc-dependent metalloendopeptidase, structurally similar to ACE and ACE2 (Santos et al., 2019), is known to degrade amyloid-β precursor protein (APP). *THOP1* expression level elevates, particularly in cortical regions, in mouse models of Alzheimer’s disease (Climer, 2025) (Fig. 2B). A notable finding is the prominent role of aminopeptidases in the RAS pathway. While *ENPEP* and *ANPEP* (*AP-N*) genes appear to be predominantly associated with the RAS, IRAP was involved in nearly all pathways analyzed, except for vasopressin-regulated water reabsorption. Our bioinformatics analysis also revealed a strong interaction between IRAP and the proteins Ywha(s), members of the 14-3-3 protein family (Tyrosine 3-Monooxygenase/Tryptophan 5-Monooxygenase Activation Proteins) (Franz et al., 2018), suggesting a potential regulatory complex influencing RAS signaling and its intersection with other physiological pathways (Fig. 2C). Through their encoded 14-3-3 proteins, these interactions are hypothesized to modulate RAS signaling by influencing AGTR1 downstream pathways, endothelial nitric oxide synthase (eNOS), aldosterone synthesis, and renin release (Kraehling and Sessa, 2015). These findings underscore the potential role of 14-3-3 proteins as critical modulators of cardiovascular function and blood pressure regulation. Further investigation is necessary to validate these findings.

### 3.3. Protein expression and pathway enrichment in the hypertensive mouse model

**Fig. 3.**
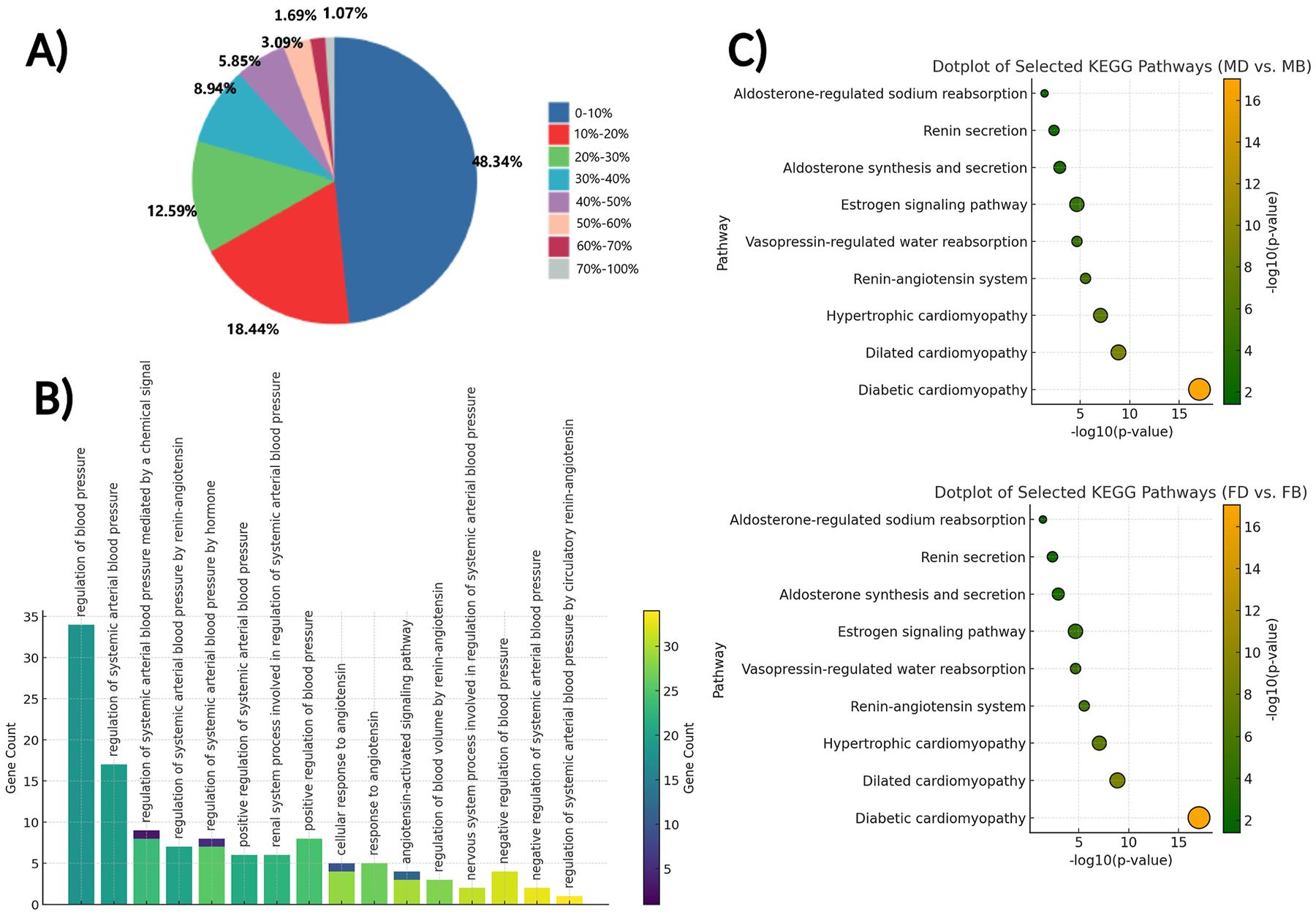
Pathway enrichment and functional annotation of blood pressure regulatory genes. (**A**) Pie chart representing the distribution of genes across different blood pressure regulatory categories. Each slice indicates the percentage of genes involved in specific functions, with the largest proportion (48.34 %) participating in 0–10 % of categorized regulatory roles. (**B**) Bar plot showing gene ontology (GO) enrichment analysis of genes involved in blood pressure regulation. The x-axis displays GO terms related to blood pressure regulation, while the y-axis shows the number of genes associated with each term. Bars are color-coded by gene count. (**C**) Dot plots representing the KEGG pathway enrichment analysis of differentially expressed genes in male (MD vs. MB, top panel) and female (FD vs. FB, bottom panel) DOCA-salt hypertensive models compared to their respective controls. The x-axis represents the −log_10_(p-value), indicating pathway enrichment significance. Dot size reflects gene count per pathway, and color scale corresponds to enrichment scores. Pathways associated with the RAS and its related pathways are prominently enriched in both sexes.

The total statistics for the mass spectrometry database search results and the number of identified proteins were as follows: the female hypertensive plasma samples (FD) identified 1376 proteins, female control plasma samples (FB) identified 1455 proteins, male hypertensive plasma samples (MD) identified 1235 proteins, and male control plasma samples (MB) identified 1331 proteins. In mass spectrometry–based proteomic analysis, each protein is identified by detecting its constituent peptides. A higher number of matching peptides increases confidence in protein identification. In our dataset, 48.34 % of the identified proteins had a sequence coverage between 0 and 10 %, indicating minimal peptide representation, while 33.22 % of proteins showed a coverage of ≥20 %, which is considered relatively robust. The average protein identification coverage was 17.56 %. Protein coverage reflects the proportion of a protein’s amino acid sequence detected through peptides and serves as a useful indicator of the reliability and completeness of the protein identification. (Fig. 3A). At the initial stage of the analysis, we performed Gene Ontology (GO) enrichment to identify biological processes linked to blood pressure. The resulting bar plot revealed sex-specific differences in gene involvement, particularly in pathways related to systemic arterial blood pressure regulation, renin-angiotensin signaling, and the response to angiotensin. (Fig. 3B). These bioinformatics analyses were conducted from two complementary perspectives to provide deeper insights into the functional attributes of these proteins and their potential relevance to the study’s objectives, facilitating the selection of key proteins for further detailed investigation. KEGG pathway enrichment analysis was employed to elucidate the biological functions of differentially expressed proteins by mapping them to regulatory pathways in the KEGG database, which provides graphical representations of the RAS and its related pathways. The statistical significance of pathway enrichment was determined using p-values, with p < 0.05 indicating significant enrichment. The dot plot results highlight the diabetic cardiomyopathy pathway due to its overlap with components of the RAS in the KEGG database. This enrichment reflects shared molecular elements rather than a direct link to diabetes or metabolic dysfunction, as the samples were not derived from a metabolic disease model. (Fig. 3C).

### 3.4. Cross-validation with in silico predictions

**Fig. 4.**
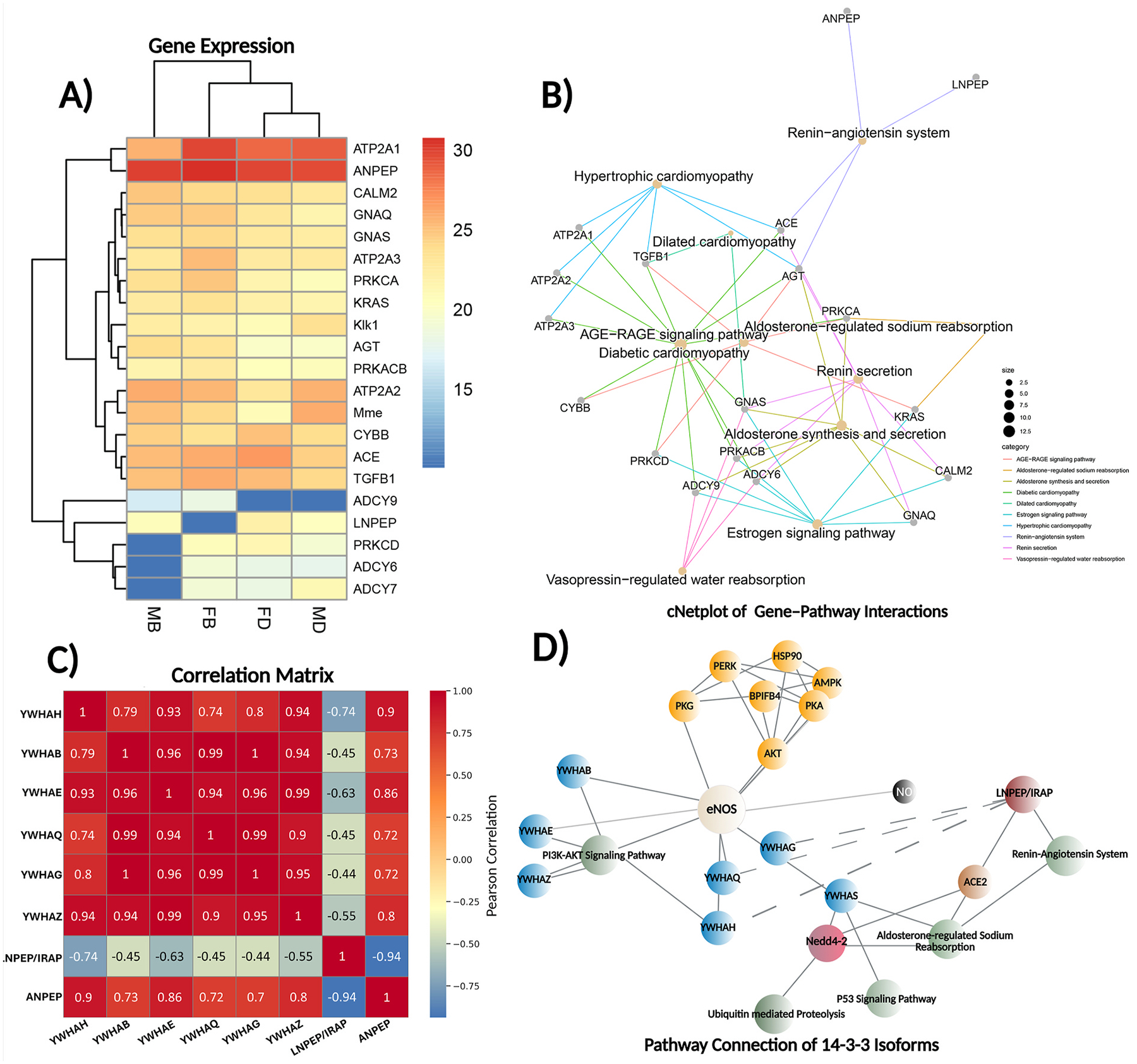
Integrated Bioinformatics and Proteomics Analysis of RAS-related Genes and Pathways in Hypertensive Mice. (A) pHeatmap displaying differential gene expression levels in male control (MB), female control (FB), female DOCA-salt hypertensive (FD), and male DOCA-salt hypertensive (MD) groups. Gene expression intensity is indicated by the color gradient (blue: lower expression, red: higher expression). (**B**) cNetplot illustrating gene-pathway interactions among identified key genes. Node size corresponds to the significance of gene involvement, while colored lines depict distinct pathways, emphasizing central genes within multiple pathways. (**C**) Correlation matrix illustrating Pearson correlations between LNPEP/IRAP, ANPEP, and isoforms of 14-3-3 proteins (Ywha(s)). Negative correlations (blue) and positive correlations (red) reveal inverse relationships among LNPEP/IRAP, ANPEP, and 14-3-3 isoforms. (**D**) Pathway network demonstrating connections among LNPEP/IRAP, ANPEP, ACE2, Ywha(s), Nedd4-2, eNOS and associated signaling pathways.

**Fig. 5.**
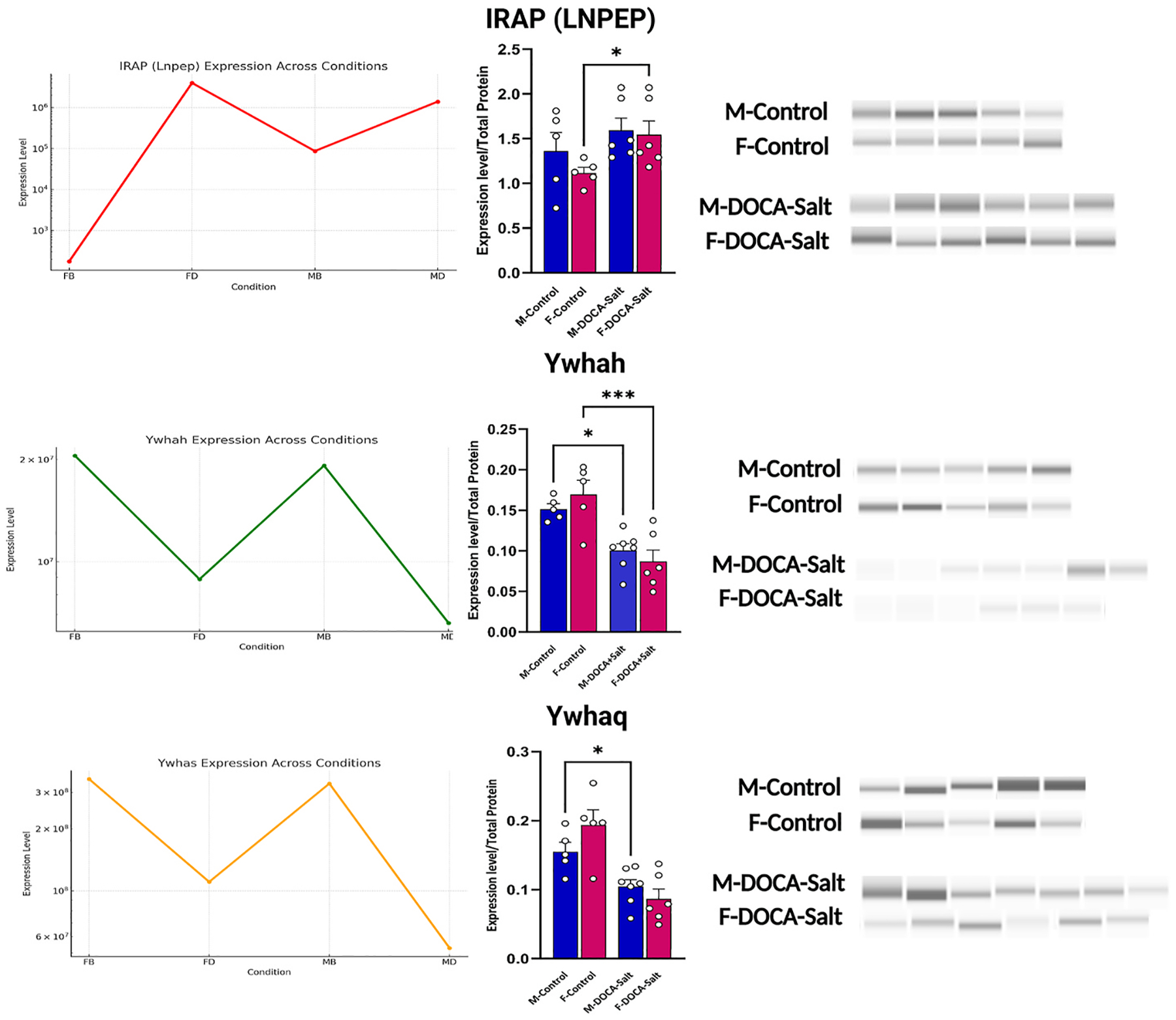
*In silico* and *ex vivo* analyses of LNPEP/IRAP, Ywhah, and Ywhaq in the context of sex-specific hypertension revealed a negative interaction between LNPEP/IRAP and the other aminopeptidases in aged DOCA-Salt hypertensive mice and their respective controls.

A total of 5690 proteins were quantified in male mice, while 5704 proteins were quantified in female mice. Among these, 2560 proteins exhibited significant changes, defined as fold change <0.71 or >2.3 with a p-value <0.05. In the subsequent step, we enriched known pathways within the RAS using their respective IDs and descriptions. Enrichment statistics were assessed in terms of p-values, adjusted p-values, and q-values. First, we aimed to identify all genes with scores exceeding 0.9 that were common to at least three pathways, alongside genes involved in the RAS based on the KEGG pathway database (map04614) in our proteomics dataset. We subsequently evaluated their expression levels in hypertensive mice compared to control conditions using pheatmap (Fig. 4A) and assessed the significance of their pathway positions using a cNetplot (Fig. 4B). Our analysis revealed altered ACE expression levels in hypertensive conditions in male and female mice. Moreover, we noted a particularly significant increase in LNPEP expression levels in hypertensive female mice compared to controls. Correlation analysis of the proteomic data across different isoforms of the 14-3-3 protein family demonstrated a negative correlation with IRAP. Furthermore, our results showed that ANPEP (AP-N) exhibits the strongest negative correlation with IRAP. Furthermore, our results showed that ANPEP exhibits the strongest negative correlation with IRAP (Fig. 4C). Bioinformatic analysis combining our findings with existing literature indicated that LNPEP negatively correlates with Ywhas. The Ywhas interacts with Nedd4-2, inhibiting its activity (Joshi et al., 2022; Pohl et al., 2021), which plays a critical role in blood pressure regulation via ACE2 ubiquitination in the RAS (Mohammed et al., 2023) (Fig. 4D). Capillary western analysis was performed to measure the expression of LNPEP, ANPEP, Ywhah and Ywhas proteins in the DOCA-Salt model thus providing valuable insights into the mechanisms underlying salt-sensitive hypertension. The DOCA-Salt model was selected based on its well-established ability to replicate key features of human hypertension, particularly volume-dependent, low-renin hypertension and vascular remodeling (Basting and Lazartigues, 2017; Xia et al., 2015). The results revealed a negative association between LNPEP and Ywhas expression, but not in Ywhah, consistent with our *in silico* and *ex vivo* findings (Fig. 5). Collectively, these results underscore the role of IRAP in hypertension and suggest that specific isoforms of the 14-3-3 protein family may interfere with hypertension-related pathways. Further investigation into these molecular alterations could provide deeper insights into the mechanisms of salt-sensitive hypertension.

## 4. Discussion

**Fig. 6.**
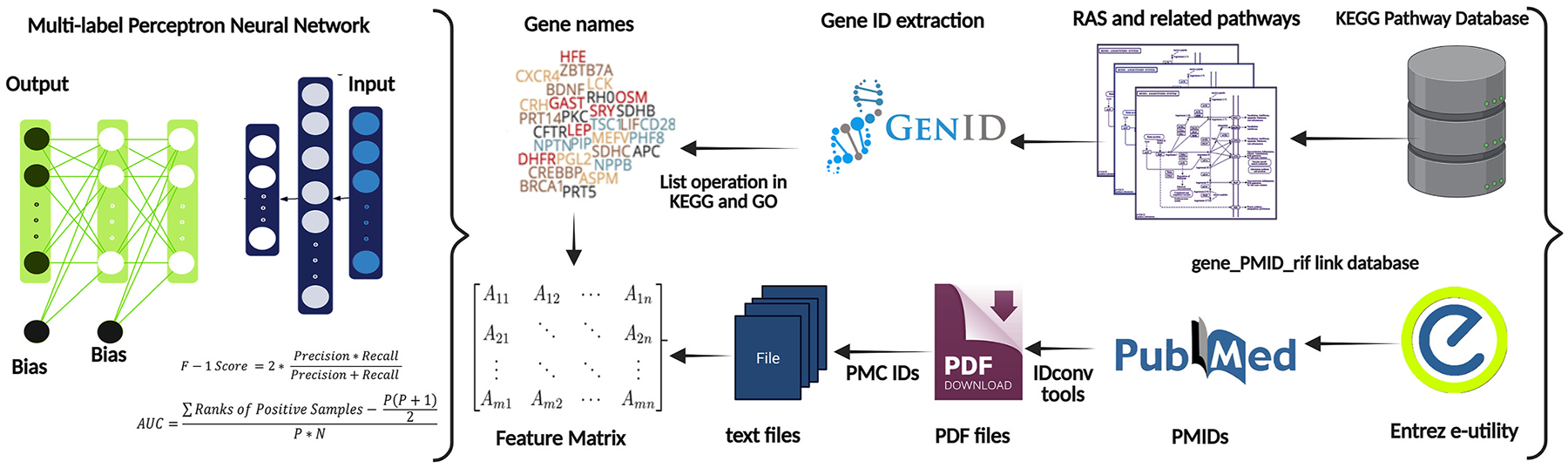
Schematic overview of the integrated bioinformatics workflow. The pipeline begins with a multi-label perceptron neural network, which is applied to a feature matrix to predict gene-pathway associations. Predicted gene names are analyzed for enrichment using KEGG and GO databases, followed by gene ID conversion via GenID. RAS-related pathways are extracted from the KEGG pathway database. To support computational predictions, literature mining is conducted using the Entrez e-utility and PubMed, linking gene IDs to PMIDs. Relevant articles are converted from PMIDs to PMC IDs and downloaded as PDF or text files for curation. This combined *in silico* and literature-based approach enhances the biological validation of key genes involved in cardiometabolic disease.

The RAS represents one of the most intricate and physiologically crucial regulatory systems in human biology, governing blood pressure regulation, fluid balance, and cardiovascular function. Despite decades of research, the full complexity of gene functions within this system remains incompletely understood, partly due to the multifunctional roles of individual components across different pathways. Our study introduces an innovative multi-label deep learning framework that systematically annotates gene functions in the RAS while experimentally validating key computational predictions through proteomic analysis in a hypertensive model. This comprehensive approach not only advances our understanding of RAS biology but also demonstrates the power of integrating artificial intelligence with traditional experimental methods in biomedical research. Recent advances in machine learning applications for biological systems have revolutionized our ability to process and interpret vast amounts of biomedical data (Castiglioni et al., 2021). However, existing approaches have been limited by their reliance on single-label classification systems, which fail to capture the pleiotropic nature of genes operating in complex biological networks like the RAS. Our model addresses this critical limitation through several innovative features. First, the incorporation of PCA before multi-label classification effectively reduces the dimensionality of our feature space while preserving the most biologically relevant information. This preprocessing step proved particularly valuable given the high dimensionality of our dataset comprising over 39,000 publications. Second, the implementation of an MLP architecture specifically optimized for multi-label classification allows our model to capture non-linear relationships between gene functions and pathway associations that would be missed by conventional methods. Fig. 6 shows a schematic overview of the integrated bioinformatics workflow.

The performance metrics of our model (ROC-AUC: 0.8697; Precision: 0.7474) compare favorably with recent deep learning applications in genomics (Weng et al., 2025) and demonstrate a substantial improvement over traditional machine learning approaches such as Random Forest and Support Vector Machine (SVM) for this specific application. Notably, our model maintained strong performance across both cross-validation and independent test datasets, indicating robust generalizability. Furthermore, the reclassification of pathways into three major branches led to improvements in performance metrics, with an enhanced ROC-AUC of 0.9182 and Precision of 0.8312 scores. These results align with emerging trends in computational biology that emphasize the importance of specialized architectures for particular biological questions (Watson, 2022). Previous research utilizing machine learning approaches has demonstrated the effectiveness of Stacked Denoising Autoencoder Multi-Label Learning (SdaMLL) in cancer genomics (Guan et al., 2018). Our computational predictions identified several key genes with particularly interesting patterns of multifunctional involvement in RAS pathways. The prominent role of *EDNRA* in blood pressure regulation pathways confirmed previous studies, serving as an important validation of our model’s predictive capabilities. In this regard, Benjafield et al. showed that there is a direct link between the *EDNRA* gene and hypertension, as the 5′-UTR of exon eight of this gene has a variant that is associated with increased systolic and diastolic blood pressure (Benjafield et al., 2003). More intriguing were the predictions regarding ESR2 involvement in hypertension pathways, which may provide mechanistic insights into well-documented but poorly understood sex differences in cardiovascular disease prevalence and progression. Consistent with the data from this study, Ogawa et al. found that variations in the *ESR2* gene could influence certain blood pressure regulators, potentially contributing to hypertension development among Japanese women (Ogawa et al., 2000). IRAP has been identified as the binding site and enzymatic target of Angiotensin IV (Ang IV), which functions as a competitive, though non-selective, inhibitor of IRAP. By binding to IRAP, Ang IV inhibits its enzymatic activity, resulting in enhanced learning, reduced ischemic damage in stroke models, and improved survival and neurological outcomes following stroke. However, due to its lack of selectivity, Ang IV may also interact with other targets, including the AGTR1 and AGTR2 as well as aminopeptidase N (Stragier et al., 2008; Telianidis et al., 2023). This enzymatic activity is implicated in the regulation of blood pressure, glucose metabolism, and overall cardiovascular function (Singh et al., 2023; Walton et al., 2020; Weiland and Verspohl, 2009). Our pathway analysis revealed IRAP’s presence across all examined pathways except for Vasopressin-Regulated Water Reabsorption, emphasizing its involvement in hypertension. The interaction between 14-3-3 proteins and eNOS plays a crucial regulatory role in maintaining vascular health, particularly under conditions related to aging and hypertension. According to Kraehling and Sessa, a longevity-associated variant of the BPIFB4 gene (I229V) is preferentially phosphorylated by the kinase PERK at serine-75. This phosphorylation event facilitates the binding of BPIFB4 to 14-3-3 proteins, which act as scaffolding molecules that retain BPIFB4 in the cytosol. The stabilized BPIFB4/14-3-3 complex subsequently recruits HSP90, a well-known activator of eNOS. Through this recruitment, HSP90 enables the phosphorylation of eNOS at serine-1177 by kinases such as Akt, AMPK, PKA, or PKG-an essential modification for enhancing eNOS activity and increasing nitric oxide (NO) production. This cascade ultimately improves endothelial function, lowers blood pressure, and contributes to vascular protection. Thus, 14-3-3 proteins indirectly enhance eNOS activation by stabilizing phosphorylated BPIFB4 and facilitating the assembly of a signaling complex that promotes NO synthesis, highlighting a sophisticated mechanism that supports cardiovascular health (Kraehling and Sessa, 2015). Our proteomics data showed an increase in IRAP levels accompanied by a decrease in Ywha(s) in EV-derived proteins, supporting the presence of this regulatory interaction. These findings may offer new insights into the mechanistic link between hypertension and its related pathways (Joshi et al., 2022; Pohl et al., 2021). Our results revealed a strong negative correlation between IRAP and several isoforms of the 14-3-3 protein family. In the context of hypertension, ubiquitination of ACE2 facilitates the recruitment of specific 14-3-3 isoforms-particularly ε,β, and γ-which in turn inhibit the binding of the Nedd4-2 E3 ubiquitin ligase (Joshi et al., 2022; Pohl et al., 2021). This inhibition leads to decreased ACE2 stability and activity, reducing the conversion of Ang II to Ang-(1–7), a critical step in blood pressure regulation (Mohammed et al., 2023). To support these observations, we conducted both *in silico* and *ex vivo* analyses, which demonstrated significant differences between hypertensive subjects and their respective controls, with the most pronounced effects seen in hypertensive female mice. While direct interactions between 14-3-3 proteins and classical components of the RAS remain to be fully elucidated, our findings suggest a potential regulatory role for the IRAP-Ywha(s) axis in blood pressure modulation through the recruitment of Nedd4-2 and ACE2. It seems that the IRAP inhibitors, and aptamers targeting 14.3.3 proteins, may offer a new paradigm for treating hypertension and cardiovascular diseases (Stevenson et al., 2008; Telianidis et al., 2023). Looking ahead, future studies should explore additional *in vivo* models-such as spontaneously hypertensive rats (SHR) or Ang II-induced hypertensive models to validate the generalizability of our *in-silico* findings. Furthermore, molecular techniques such as overexpression or knockdown of specific 14-3-3 isoforms are warranted to assess their regulatory effects on LNPEP, and consequently, on Nedd4-2 and ACE2. Most importantly, these predicted regulatory interactions must be validated in human samples to inform the development of novel strategies for managing chronic hypertension and related cardiovascular diseases. From a methodological standpoint, our study highlights the strength of integrating computational predictions with experimental validation in systems biology research. This iterative framework-where computational insights inform targeted experiments, and experimental data refine computational models-forms a virtuous cycle that accelerates discovery while upholding scientific rigor. Such an approach is particularly valuable in complex systems like the RAS, where reductionist methods may fall short in capturing the broader physiological context.

## 5. Conclusion

In this study, we developed a multi-label deep learning framework to systematically annotate gene functions within the RAS, overcoming the limitations of traditional single-label approaches. By analyzing over 39,000 biomedical publications and integrating PCA with a MLP, our model achieved superior performance (ROC-AUC: 0.9182) compared to conventional methods. This framework enabled efficient and accurate annotation of multifunctional genes and revealed key RAS-associated components such as IRAP, Ywha(s), EDNRA, and ESR2 with distinct sex-specific expression patterns. These findings significantly advance our understanding of cardiovascular physiology and highlight novel therapeutic targets for hypertension and related diseases. Experimental validation in hypertensive mice confirmed the upregulation of IRAP and its interaction with 14-3-3 proteins, implicating these components in the dysregulation of AGTR1-mediated RAS signaling pathways, including effects on endothelial nitric oxide synthase activity and aldosterone synthesis. This work provides a template for future systems biology research and AI-driven biomarker discovery, paving the way for transformative advancements in both research and clinical applications in cardiovascular disease.

## Figures and Tables

**Table 1:** Average results from the cross-validation method on the dataset based on four selected and primary metrics.

Cross-Validation	Precision (Macro)	F-1 Score (Macro)	Ranking Loss	ROC-AUC (Macro)
**SVM**	0.6233	0.3041	0.1810	0.8102
**Random Forest**	0.7900	0.1856	0.1910	0.7840
**XGBoost**	0.7228	0.2622	0.2035	0.7931
**MLP**	0.7474	0.5846	0.1096	0.8697

**Table 2:** Average results from training on the whole dataset and predicting the test set based on our primary metrics.

Test Results	Precision (Macro)	F-1 Score (Macro)	Ranking Loss	ROC-AUC (Macro)
**SVM**	0.7600	0.5033	0.1703	0.8435
**Random Forest**	0.8000	0.2645	0.1613	0.8362
**XGBoost**	0.9524	0.3279	0.1911	0.8350
**MLP**	0.6829	0.6120	0.1765	0.8528

**Table 3:** Average results from the cross-validation method on the dataset based on four selected and primary metrics with 3 categories.

Cross-Validation	Precision (Macro)	F-1 Score (Macro)	Ranking Loss	ROC-AUC (Macro)
**SVM**	0.6946	0.6156	0.1391	0.8420
**Random Forest**	0.7956	0.6838	0.1147	0.8903
**XGBoost**	0.7891	0.6372	0.1383	0.8602
**MLP**	0.8312	0. 8058	0.0963	0.9182

**Table 4:** Average results from training on the whole dataset and predicting the test set based on our primary metrics with 3 categories.

Test Results	Precision (Macro)	F-1 Score (Macro)	Ranking Loss	ROC-AUC (Macro)
**SVM**	0.6429	0.5854	0.2062	0.7561
**Random Forest**	0.7692	0.6723	0.1187	0.8689
**XGBoost**	0.7917	0.6609	0.1313	0.8720
**MLP**	0.7867	0.7919	0.1076	0.8827

**Table 5:** RAS is involved in at least ten confirmed pathways.

	Related pathways	Most Important Gene (Max predicted probabilities)	Free full text
**Hypertension-related pathways**	Renin-angiotensin system (RAS)	***AGTR2*** *, PRCP, ANPEP, ENPEP, ATP6AP2, AGTR1, KLK1/2, NLN, MAS1*	8285
	Renin secretion (RS)	***EDNRA****, PTGER4, PTGER2, CALML3, PPP3CA, PDE3A, ADCYAP1, PPP3R1, GUCY1A1, ADRB3*,	7658
	Aldosterone synthesis and secretion (AS)	***CYP11A1****, CYP11B2, CYP21A2, PRKD2, LIPE, PRKD1, SCARB1, CAMK4, CALML3, ITPR3*,	4473
	Aldosterone-regulated sodium reabsorption (ARSR)	***SFN/Ywhas****, SGK1, KCNJ1, NEDD4L, SCNN1B, PDPK1, FXYD2, SCNN1A, HSD11B2, SCNN1G*,	931 (258)
	Estrogen signaling pathway (ESP)	***ESR2****, FKBP4, FKBP5, GPER1, GRB2, HSPA1A, HSPA8, HSP90AA1, KRT10, SHC3*,	4901
	Vasopressin-Regulated Water Reabsorption (VRWR)	***AQP3****, AQP4, ARHGDIB, AVPR2, AQP2, DYNLL1, DCTN4, DYNC1H1, DCTN6, NSF*,	498 (84)
**Diabetes-related pathways**	Diabetic cardiomyopathy (DiCM)	***ADAR****, NLRP3, CGAS, CSF2, CFD, FAU, FCGR2A, FGA, TBK1, STING1*,	2858 (60)
	AGE-RAGE signaling pathway in diabetic complications (AGE-RAGE)	***CDK4****, CDKN1B, COL4A1, COL4A2, COL4A5, AGER, FOXO1, FN1, NOX1, ICAM1*,	1278
**Cardiac disease-related pathways**	Hypertrophic cardiomyopathy (HCM)	***ATP2A2*,** *SGCG, TNNC1, TPM1, TPM3, TTN, ACTC1, LAMA1, SGCE, SLC8A1*	2217 (28)
	Dilated cardiomyopathy (DCM)	***DAG1****, DES, DMD, EMD, LAMA1, ITGA2B, ITGB1, ITGB3, LMNA, MYBPC3*,	6382 (52)

## Data Availability

Data are available via ProteomeXchange with identifier PXD068025.

## Abbreviations

RAS: Renin-angiotensin system
PCA: Principal Component Analysis
MLP: Multi-Layer Perceptron
EV: Extracellular vesicle
Ang-II: Angiotensin-II
AGTR1: Angiotensin-II type 1 receptor
LNPEP: Leucyl/cysteinyl aminopeptidase
IRAP: Insulin-regulated aminopeptidase
SVM: Support Vector Machine
OvR: One-vs-Rest
ADCY: Adenylate cyclases
CALM: Calmodulin
EDNRA: Endothelin Receptor Type A
ESR2: Estrogen Receptor Beta
SFN: Stratifin/Ywhas/14-3-3σ
ARSR: Aldosterone-Regulated Sodium Reabsorption
CMA1: Chymase 1
eNOS: Endothelial nitric oxide synthase
SdaMLL: Stacked Denoising Autoencoder Multi-Label Learning
